# Quantitative proteomics of heat-treated human cells show an across-the-board mild depletion of housekeeping proteins to massively accumulate few HSPs

**DOI:** 10.1007/s12192-015-0583-2

**Published:** 2015-04-08

**Authors:** Andrija Finka, Vishal Sood, Manfredo Quadroni, Paolo De Los Rios, Pierre Goloubinoff

**Affiliations:** 1 Department of Plant Molecular Biology, Faculty of Biology and Medicine, University of Lausanne, 1015 Lausanne, Switzerland; 2 Laboratoire de Biophysique Statistique, School of Basic Sciences, Ecole Polytechnique Fédérale de Lausanne (EPFL), 1015 Lausanne, Switzerland; 3 Department of Biochemistry, Faculty of Biology and Medicine, University of Lausanne, 1015 Lausanne, Switzerland

**Keywords:** hsp70, Hsc70, Hsp90, Molecular chaperone, Heat shock, Jurkat cells

## Abstract

Classic semiquantitative proteomic methods have shown that all organisms respond to a mild heat shock by an apparent massive accumulation of a small set of proteins, named heat-shock proteins (HSPs) and a concomitant slowing down in the synthesis of the other proteins. Yet unexplained, the increased levels of HSP messenger RNAs (mRNAs) may exceed 100 times the ensuing relative levels of HSP proteins. We used here high-throughput quantitative proteomics and targeted mRNA quantification to estimate in human cell cultures the mass and copy numbers of the most abundant proteins that become significantly accumulated, depleted, or unchanged during and following 4 h at 41 °C, which we define as mild heat shock. This treatment caused a minor across-the-board mass loss in many housekeeping proteins, which was matched by a mass gain in a few HSPs, predominantly cytosolic HSPCs (HSP90s) and HSPA8 (HSC70). As the mRNAs of the heat-depleted proteins were not significantly degraded and less ribosomes were recruited by excess new HSP mRNAs, the mild depletion of the many housekeeping proteins during heat shock was attributed to their slower replenishment. This differential protein expression pattern was reproduced by isothermal treatments with Hsp90 inhibitors. Unexpectedly, heat-treated cells accumulated 55 times more new molecules of HSPA8 (HSC70) than of the acknowledged heat-inducible isoform HSPA1A (HSP70), implying that when expressed as net copy number differences, rather than as mere “fold change” ratios, new biologically relevant information can be extracted from quantitative proteomic data. Raw data are available via ProteomeXchange with identifier PXD001666.

**Electronic supplementary material:**

The online version of this article (doi:10.1007/s12192-015-0583-2) contains supplementary material, which is available to authorized users.

## Introduction

A mild rise of ambient temperature directly causes a mild increase in the fluidity of biological membranes, which readily translates in eukaryotes into the specific activation of heat-sensory Ca^2+^ conducting channels (Anckar and Sistonen 2011; Bromberg et al. 2013; Finka et al. 2012). Within seconds, heat initiates a heat-shock signaling cascade involving Ca^2+^-activated calmodulin kinases, which in turn activate the heat-shock transcription factor-1 (HSF1) (Bromberg et al. 2013; Kiang et al. 1994; Voellmy and Boellmann 2007). The ensuing binding of activated HSF1 to specific promoter regions of genes encoding by definition for heat-shock proteins (HSPs) typically leads to the massive accumulation, within half an hour, of new HSP messenger RNAs (mRNAs) (Finka et al. 2011; Kline and Morimoto 1997). The resulting synthesis and accumulation, within hours, of dozens of HSPs in the various cellular compartments (Ritossa 1962; Tissieres et al. 1974) ultimately leads to a transient phenotype called acquired thermotolerance, allowing all organisms to survive short exposures, typically several hours, to otherwise lethal doses of noxious elevated temperatures (Finka et al. 2012; Saidi et al. 2009; Stevenson et al. 1987).

Classic pulse-chase experiments with ^35^S-methionine have shown that heat shock (HS) generally causes cell growth arrest and the slowing down of the steady-state synthesis of most housekeeping proteins, such as the ribosomal proteins, while concomitantly, a few specific HSPs accumulate massively (Lewis et al. 1975; Tissieres et al. 1974; Warner and Gorenstein 1977). In all organisms, the successful accumulation of HSPs in response to a prior mild “priming” heat shock is a precondition to the successful onset of acquired thermotolerance (Horowitz 2007; Larkindale and Vierling 2008).

The most abundant HSPs belong to conserved families of molecular chaperones, principally the HSP70/110, HSP100, HSP90, HSP60, and the small HSPs. Yet, many nonchaperone genes, such as genes encoding for detoxifying enzymes of reactive oxygen species, may also become massively expressed under heat shock (Finka et al. 2011). During heat stress, molecular chaperones can prevent protein aggregation. Then, after the stress, chaperones can actively drive unfolding/refolding of the damaged polypeptides to their native state or target them to degradation (Diamant and Goloubinoff 1998; Goloubinoff et al. 1997; Mattoo and Goloubinoff 2014; Priya et al. 2013; Rampelt et al. 2012).

In the human genome, about 170 genes have been bio-informatically identified as members of the “chaperome” network on the basis of their sequence homologies with members of the conserved core chaperone families HSP70/HSP110s, HSP100s, HSP90s, HSP60/CCT, and sHSPs, with conserved co-chaperones, and with folding enzymes, such as the peptidyl prolyl isomerases and the protein disulfide isomerases (Finka et al. 2011). Compared to their frequency in the genome, these “chaperome” members were found to be 20 times more likely to be also heat inducible. Yet, noticeably, the mRNA levels of more than two thirds of the chaperome members remain virtually unchanged during heat shock and some of them may correspondingly be called heat-shock cognates (HSCs) (Finka et al. 2011). Thus, the cytosolic chaperone HSPA8 (HSC70) (Finka and Goloubinoff 2014), which can be as much as 1 % of the total protein mass in eukaryotes, is generally thought to be evenly expressed under stress and physiological conditions. In contrast, HSPA1A (HSP70), which shares 90 % sequence identity with HSPA8 and is virtually absent from unstressed noncancerous cells, is massively expressed during and following various chemical and abiotic stresses, such as heat shock (Ingolia et al. 1982).

In unstressed human cells, the members of the chaperome network may sum up into 10 % of the total protein mass, where they act as key regulators of cellular proteostasis (Finka and Goloubinoff 2013; Kikis et al. 2010). Chaperones assist the de novo folding of nascent polypeptides, serve as translocating/pulling and unfolding motors of polypeptides across cellular compartments, regulate protein activity, control vesicular trafficking, and can mediate lysosomal and proteasomal protein degradation (Cassina and Casari 2009; Dittmar et al. 1997; Eisenberg and Greene 2007; Finka and Goloubinoff 2013; Goloubinoff and De Los Rios 2007; Kalia et al. 2011; Rampelt et al. 2012; Schuermann et al. 2008).

The cellular response to a mild temperature increase has been studied at the protein level using classic analytic approaches such as pulse-labeling with ^35^S-methionine and semiquantitative Western blots (Tissieres et al. 1974) and at the mRNA level with northern blots, RT-PCRs, and DNA microarrays (Didomenico et al. 1982; Laramie et al. 2008; Wang et al. 1999). These methods, which typically provided relative values expressed in “fold change” of HSP amounts after the stress as compared to before the stress, lacked essential biological information about the absolute concentrations and copy numbers per cell of each HSP, during and following stress. When expressed as relative values, the amounts of HSP mRNA transcripts in mammalian cells were often found to be ten- to hundredfolds higher after HS than before HS (Finka et al. 2011), whereas semiquantitative Western blots typically showed mere 2- to 4-fold increases of the corresponding HSPs, suggesting that for unclear reasons, the translation efficiency of the HSP mRNAs under heat stress is dramatically modified (Kiang et al. 1994, 2000).

Here, we used stable isotope labeling with amino acid in culture (SILAC) and high-resolution mass spectrometry (MS) to estimate in the human Jurkat T-cell line the relative mass and absolute copy number per constant cell volume of the most abundant HSPs at 37 °C and during and following 41 °C treatment. In parallel, we also used NanoString technology to quantify a limited set of HSP and non-HSP mRNAs and express them in copy numbers per cell (Geiss et al. 2008). HS caused a general across-the-board mild depletion of many abundant housekeeping proteins apparently owing to the slowing down of their replenishment by translation on fewer ribosomes, which became transiently recruited by excessive new HSP mRNAs. A similar effect was observed in cells treated isothermally with an HSP-inducing inhibitor of Hsp90. Absolute differences of copy numbers per cell provided new information about the possible biological function of HSPA8 as compared to HSPA1A/B.

## Material and methods

### SILAC labeling and heat shock of cells

All isotope-labeled amino acids were from Cambridge Isotope Laboratories (CIL), Andover, MA, USA. SILAC cell culture media were from Pierce/Thermo Scientific. Sequencing grade modified trypsin was from Promega. Protease and phosphatase inhibitors were from Roche Biochemicals. All other chemicals were of the highest grade available from Sigma-Aldrich.

SILAC labeling was performed essentially as described (Fierro-Monti et al. 2013). Briefly, Jurkat T lymphocytes were cultured in RPMI 1640 medium with 10 % (*v*/*v*) dialyzed FBS for all experiments. Isotope-labeled (^13^C_6_^15^N_4_-L-arginine and ^13^C_6_-L-lysine) or light amino acids were added at a final concentration of 100 mg/l, while proline was at 180 mg/l. Labeling of the “heavy” culture was tested beforehand and was >98 %. For heat shock, subconfluent flasks of cells (one per condition, time point, and replicate) were transferred at experiment time *t* = 0 from the 37 °C incubator to either a water bath at 37 °C or an identical water bath kept at 41 °C. After 4 h at 41 °C, cell flasks to be used for the *t* = 6 and 10 h time points were returned to the incubator at 37 °C. Viability of cells was examined at *t* = 10 h and at *t* = 24 h and found to be very high (>95 %) for both control and heat-shocked cells, although counts were generally lower for heat-shocked cells.

### Protein extraction and digestion

Cells at *t* = 2, 4, 6, and 10 h were harvested by centrifugation, washed three times with ice-cold PBS, and lysed by sonication in 10 mM Tris, 8 M urea, and protease and phosphatase inhibitors. After centrifugation, protein concentrations were determined by gel electrophoresis, Coomassie blue staining, and densitometry, compared against a prequantified standard cell extract. Equimolar extracts from light/heavy labeled cells were combined, reduced with 5 mM DTT, alkylated with 20 mM iodoacetamide, and precipitated with 5 % trichloroacetic acid and 1 % (*w*/*v*) sodium deoxycholate. Proteins were resuspended in 8.0 M urea in 50 mM triethylammonium bicarbonate buffer (pH = 8) by sonication, diluted with buffer to reach 2.0 M urea, and digested overnight with trypsin (1:100 *w*/*w* ratio). The obtained peptide mixtures (200 μg total material) were desalted on SepPak C18 cartridges (Waters Corp., Milford, MA), dried, and subsequently fractionated into 6 pH fractions as described (Wisniewski et al. 2009), with the difference that a strong cation exchange resin (POROS HS, Life Technologies) was used and thus peptides were loaded in acidic conditions.

### Mass spectrometry analysis

Desalted aliquots of the fractionated digests were analyzed on a hybrid linear trap LTQ-Orbitrap Velos mass spectrometer (Thermo Fisher, Bremen, Germany) interfaced via a nanospray source to a Dionex RSLC 3000 nanoHPLC system (Dionex, Sunnyvale, CA, USA). Peptides from all fractions were separated on a reversed-phase Acclaim PepMap nanocolumn (75 μm ID × 25 cm, 2.0 μm, 100 A, Dionex) at 0.3 μl/min with a gradient from 5 to 45 % acetonitrile in 0.1 % formic acid (total time 140 min). Full MS survey scans were performed at 60,000 resolution. In data-dependent acquisition controlled by Xcalibur 2.0.7 software (Thermo Fisher), the 20 most intense multiply charged precursor ions detected in the full MS survey scan were selected for collision-induced dissociation (CID) fragmentation in the LTQ linear trap and then dynamically excluded from further selection during 120 s. The window for precursor isolation was of 3.0 *m*/*z* units around the precursor.

### MS data analysis: identification and quantification

MS data were analyzed and quantitated using MaxQuant version 1.3.0.5, which incorporates the Andromeda search engine (Cox et al. 2011). The database used was the 2012_02 release of the human reference proteome from UniProtKB containing 81,213 protein sequences (Apweiler et al. 2014). Cleavage specificity was trypsin (cleavage after K, R; no cleavage at KP, KR) with two missed cleavages. Mass tolerances were of 7 ppm for the precursor and 0.5 Da for CID tandem mass spectra. The iodoacetamide derivative of cysteine was specified as a fixed modification, and oxidation of methionine and protein N-terminal acetylation were specified as variable modifications. Protein identifications were filtered at 1 % false discovery rate (FDR) established by MaxQuant against a database of reversed sequences. A minimum of one unique peptide was necessary to discriminate sequences, which shared peptides. Sets of protein sequences, which could not be discriminated based on identified peptides, were listed together as protein groups and are fully reported in the Supplementary tables. Only unique and “razor” peptides were considered for protein quantitation of sequences with shared peptides. Details of peak quantitation, normalization, and protein ratio computation by MaxQuant are described elsewhere (Cox and Mann 2008). All proteins with quantitated values (minimum evidence count = 1) were initially retained to be subjected to filtering in subsequent steps (see below). Intensity-based absolute quantification (iBAQ) parameter values (Schwanhausser et al. 2011) were also calculated by the MaxQuant software. Data on identification and quantification of individual proteins and peptides are reported in Supplementary Tables S1.

### Biostatistical analysis

The raw iBAQ values were obtained for HS and non-HS Jurkat cell lines in biological triplicates. To normalize the iBAQ linear values, each of them was multiplied by corresponding molecular weight of the polypeptide and they were summed up (in each column) to obtain the total (100 %) iBAQ-derived protein mass fraction per cell. The individual protein mass fractions were converted into individual protein copy numbers by multiplying individual protein mass fractions with estimated protein mass per cell of 81 pg and Avogadro’s constant and divided by the specific molecular weight of each polypeptide. The statistical analysis was performed with the R-statistical package.

### Nanostring/nCounter analysis

The total RNA extracted from 10^6^ untreated or HS-treated cells was isolated using the RNeasy kit [Qiagen (Valencia, CA) catalog no. 79216], purified by cold ethanol precipitation. RNA was quantified by Nanodrop and its integrity was verified by Bioanalyzer; 100 ng of RNA was hybridized overnight at 65 °C with the selected capture and reporter probes and treated according to the nCounter recommended protocol. Target/probe complexes were immobilized in nCounter cartridges for data collection using an nCounter digital. The data were analyzed according to the manufacturer’s guidelines (Supplementary Table 5).

### Estimation of the mRNA copy numbers per cell

We calculated the copy numbers of most ribosomal proteins and obtained about two million ribosomes per Jurkat cell at 37 °C. This corresponded to 7.75 pg of ribosomal RNA (rRNA) per cell according to the following calculations: 2 × 10^6^ × (7156 of rRNA bases in a human ribosome) × (333 g mol^−1^ per base representing average MW of a ribosomal base) / (6.023 × 10^23^ mol^−1^) = 7.75 pg rRNA per cell. The total amount of RNA in an average human cell ranges between 10 and 30 pg (Russo and Russo 2014) estimated to be distributed in 85 % rRNA, 10–12 % tRNA, and 2–5 % mRNA (Lodish 2000). Thus, we had 9.11 pg total RNA per cell, which was in good agreement with the 10 pg of total RNA that we used in our estimation of individual mRNA copy numbers using the Nanostring measures. Regarding mRNA quantification, an average of 300,000 mRNA copies has been found in various human tissues (Velculescu et al. 1999). Given that the mean polypeptide mass in the human proteome is 55 kDa, corresponding to mRNAs of about 2000 bp (including noncoding regions), this yields a predicted 0.29 pg of mRNA per cell, corresponding to the expected value of 3 % mRNA of the total RNA in the cell. Independent measures by serial expression gene analysis in various human tissues provided copy number for 22 identified genes, 19 of which we found to be in the same range of copy numbers (Velculescu et al. 1999).

### Data availability

Raw data and the MaxQuant output are available via ProteomeXchange (http://proteomecentral.proteomexchange.org) with identifier PXD001666.

## Results

### Conversion of the raw iBAQ values into protein mass fraction and copy number per cell

**Fig. 1:**
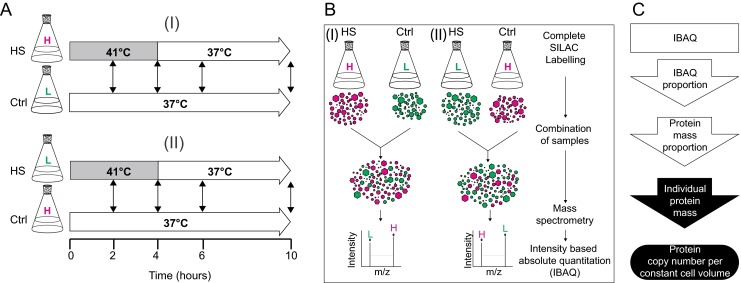
Experimental setup to identify and quantify proteins in heat-treated cells. **a** Two independent biological replicates (I) of heavy stable isotope labeling by amino acids (*SILAC*) in Jurkat cell cultures (*H*, *magenta*) for proteomic analysis were submitted to a mild heat shock (HS) at 41 °C during up to 4 h (*gray bar*) and then further incubated at 37 °C during up to 6 h (*white arrow*). The light SILAC cells (*L*, *green*), which where the control (Ctrl) samples, were kept at 37 °C (Ctrl). One swapped biological replicate (II) of light SILAC was heat shocked during 4 h at 41 °C, followed by an incubation at 37 °C for 6 h, whereas of heavy SILAC served as the controls kept at 37 °C. Cell samples were collected at 2, 4, 6, and 10 h of the experiment (*double arrows*). **b** Proteins from the HS-treated heavy and Ctrl light cells (I) and from the swapped HS-treated light and Ctrl heavy cells (II) were independently combined, trypsin digested, and quantified by mass spectrometry. The relative intensities and spectra of increased or decreased proteins were collected. **c** The resulting iBAQ numbers for individual proteins were converted into relative protein mass fractions of the total cellular proteins and further into absolute copy numbers per constant cell, as described in Finka and Goloubinoff (2013)

We generated high-throughput proteomic MS data at medium depth, to address the net changes in the concentrations of the main proteins in cultured human Jurkat cells, before and following 2 and 4 h HS (at 41 °C) and also 2 and 6 h after the end of HS. At all time points, heat-treated samples were paired with unchallenged cell cultures maintained at 37 °C as controls (Fig. 1). Three independent biological replicates were collected for each time point and temperature. We thus identified 4585 different proteins in a total of 24 independent biological samples (Supplementary Table 1), composed of twelve 41/37 °C pairs. After data filtering, this resulted in eight lists of 3164 proteins, four for each time point of the HS experiment (Supplementary Table 2).

To estimate the coverage of this medium-depth analysis, our data was compared to an earlier high-depth proteomics data in which 11,731 different proteins were identified in at least 1 of 33 independent biological samples from 11 different human tumor cell lines (Geiger et al. 2012). The sum of masses of these 11,731 proteins was previously estimated to be a good proxy for the total (~100 %) protein mass of a human cell (Finka and Goloubinoff 2013). Using the Bradford colorimetric assay, we next found that 10,000 visually counted Jurkat cells contained a total of 900 ng protein, implying 90 pg per Jurkat cell. Given a cellular diameter of 12 μm (BNID 108901; www.bionumbers.hms.harvard.edu) and a consequent average cellular volume of ~900 μm^3^, this corresponded to a protein density of 100 mg/ml that was well in the range of previous estimates of protein crowding values in various human cell types (Finka and Goloubinoff 2013; Milo 2013). Compared with the protein list for Jurkat cells of Geiger et al. (2012), the protein mass from our medium-depth dataset accounted for 90 % of the total cellular proteins, and the sum of all the protein masses in each of our protein lists was thus estimated to be 81.3 ± 0.2 pg per cell (Supplementary Table 2). We then expressed the individual iBAQ values for each identified protein in the 24 samples, as a fraction of the total (90 pg) protein mass of a typical Jurkat cell (Supplementary Table 3A). Knowing their molecular weight, copy number values per cell were obtained for each detected polypeptide (Supplementary Table 3B). From within these protein lists, the median copy number of the ribosomal proteins was 2 × 10^6^ per (904 μm^3^) Jurkat cell, a ribosomal density that matched very well earlier estimates of ribosomes being 5–10 % to the total protein mass of typical human cells (Duncan and Hershey 1983), corresponding to 4.5–9 pg ribosomal proteins, hence to 1.6–3.2 × 10^6^ ribosome per Jurkat cell (Finka and Goloubinoff 2013). Moreover, independent estimates of the 18S and 28S rRNA amounts by qPCR and fluorimetric methods indicated the presence of two to three million ribosomes per human cell (Duncan and Hershey 1983; Hollenhorst et al. 2004). This confirmed the validity of our normalization method of converting raw iBAQ values into mass fractions and copy numbers.

### HS causes an across-the-board mild depletion of many housekeeping proteins counterbalanced by a massive synthesis of few HSPs

**Fig. 2:**
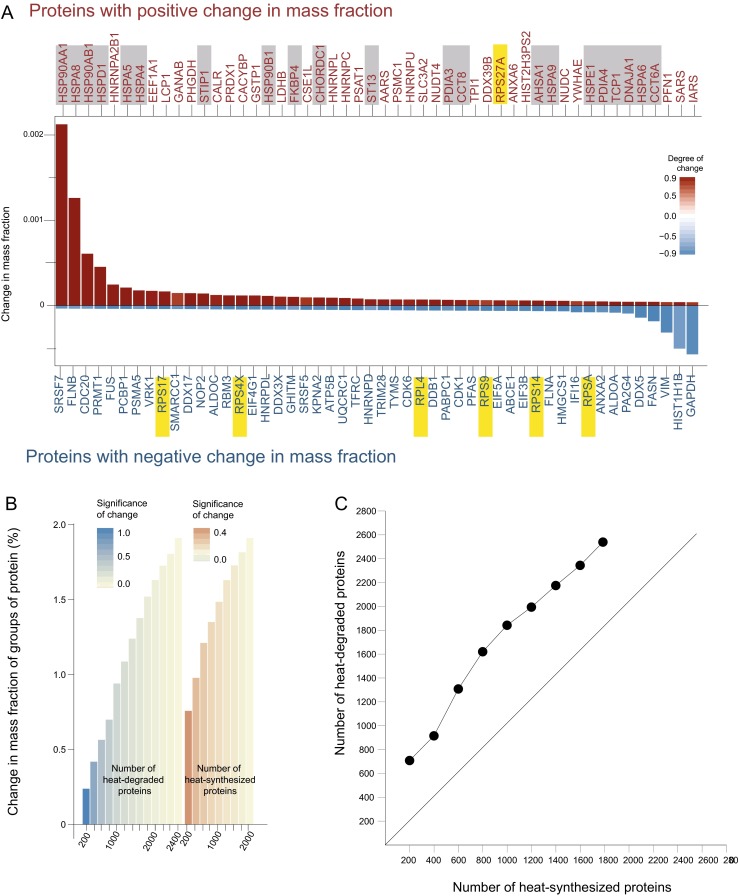
Synthesis of across-the-board protein depletion balances of few heat-accumulated proteins. **a** Mean net significant change in mass fraction (%) of the 50 most heat-accumulated (*red*) and of the 50 most heat-depleted proteins (*blue*) at all time points. Chaperome proteins are *boxed in gray* and ribosomal proteins in *yellow*. The significance scale in **a** and **c** (*dark color*, high significance; *light color*, low significance) is (1—*p* value) multiplied either by a *positive sign* (*red*) or *negative sign* (*blue*), respectively, for positive and negative changes of mass fraction. **b** *Bar graphs* showing net increments and losses of cumulative mass fraction changes in groups of analyzed proteins during HS. The heat-accumulated and heat-depleted proteins were independently computed from the sum of the dataset at all time points and ordered according to their increasing *p* values. The most *colored bar* were the sum of net mass gain (*deep orange*) or loss (*deep blue*) of the 200 proteins with the highest significance in their mass change values. The *last bars with the lightest coloring* were the sum of all heat-accumulated or heat-depleted proteins, which included also mass changes in proteins with a low degree of significance. **c** At all time points, a few (200) heat-accumulated proteins apparently gain in mass, while many more (600) are mildly heat-depleted proteins. This trend was maintained when comparing larger populations of protein with less significant mass differences. The straight median in the theoretical distribution of equal mass gains and losses was the number of heat-degraded proteins equal to the number of heat-accumulated proteins

We next addressed the possible subtle mass variations of individual proteins in response to 4 h heat treatment at 41 °C, a mild non-noxious HS temperature corresponding to a physiological fever, followed by a 6 h recovery at 37 °C. Visual observations confirmed earlier reports (Komata et al. 2004) that cell growth was arrested during HS. Growth slowly resumed only 2 and 6 h thereafter. As individual proteins showed different kinetics of synthesis and degradation, we determined the significant changes in the mass fraction of individual proteins at each of the time point (Supplementary Fig. 1) and for each protein summed all its net changes in mass fraction observed at all of the time points during and after HS (Fig. 2a). This revealed that the 50 proteins that became significantly most accumulated included 21 chaperome members (Fig. 2a, gray), which, compared to their relative representation in the human genome, was a 60-fold enrichment. In contrast, no chaperome members and six ribosomal proteins were among the 50 most heat-depleted proteins, a 35-fold enrichment in ribosomal proteins in this category (Fig. 2a), as compared to only one ribosomal protein that was found among the heat-accumulated proteins (Fig. 2a, yellow), indicating a general loss of up to 10 % of the ribosomal protein synthesis ability.

We next attempted to compare the global protein mass gains and mass losses at particular time points of the HS. Yet, because most individual values did not pass the stringent statistical thresholds, we combined the data from all time points, 2 and 4 h HS and for 2 and 6 h after the HS, and globally analyzed them as a sum (Supplementary Fig. 2). The net differences of mass fractions of the most heat-accumulated proteins (at all time points) and of the most heat-depleted proteins were then sorted in cumulative bins of 200 proteins, sorted according to their statistical significance (Supplementary Fig. 2 and Fig. 2b). Hence, the first bins (the strongest colored bars) contained the sum of the most significant differences in mass fraction in the 200 most heat-accumulated proteins that respectively summed into a mass increment of 0.75 % (Fig. 2b, right) and of most heat-depleted proteins that summed into a mass loss of 0.25 % (Fig. 2b, left). The next bin contained the sum of the net differential masses in the 400 most heat-accumulated and most heat-depleted proteins, which showed a greater mass contribution of the HSPs (0.96 %) compared to the heat-depleted proteins (0.45 %), albeit these values were with a lesser degree of statistical confidence. Adding more bins with HS-accumulated and HS-depleted proteins saturated toward a maximal of 2 % of mass change, indicating that the maximal protein mass gain by few HSPs was around 2 %, which was compensated by a smaller total mass loss, unless a greater number of heat-depleted proteins was included in the calculation. Hence, the number of mildly depleted proteins was always much larger than the number of massively accumulated HSPs to reach an equal mass gain and mass loss (Fig. 2c). The cumulative protein mass increase associated to the 200 most significantly upregulated proteins was 0.75 % of the total cellular proteins, whereas the contributions of the 720 most heat-depleted proteins was needed to reach an equivalent mass loss of 0.75 % (Fig. 2c). Similarly, the sum of the 400 most significantly upregulated proteins was 0.96 %, whereas 910 of the most heat-depleted proteins were needed to reach the same extent of mass loss. Given that no change was observed in the average size of individual cells during the HS, this implied that the strong increase of mass in few HSPs was compensated by a reciprocal across-the-board mild mass depletion in many more proteins.

### Gene ontology analysis of HS-accumulated and HS-depleted proteins

**Fig. 3:**
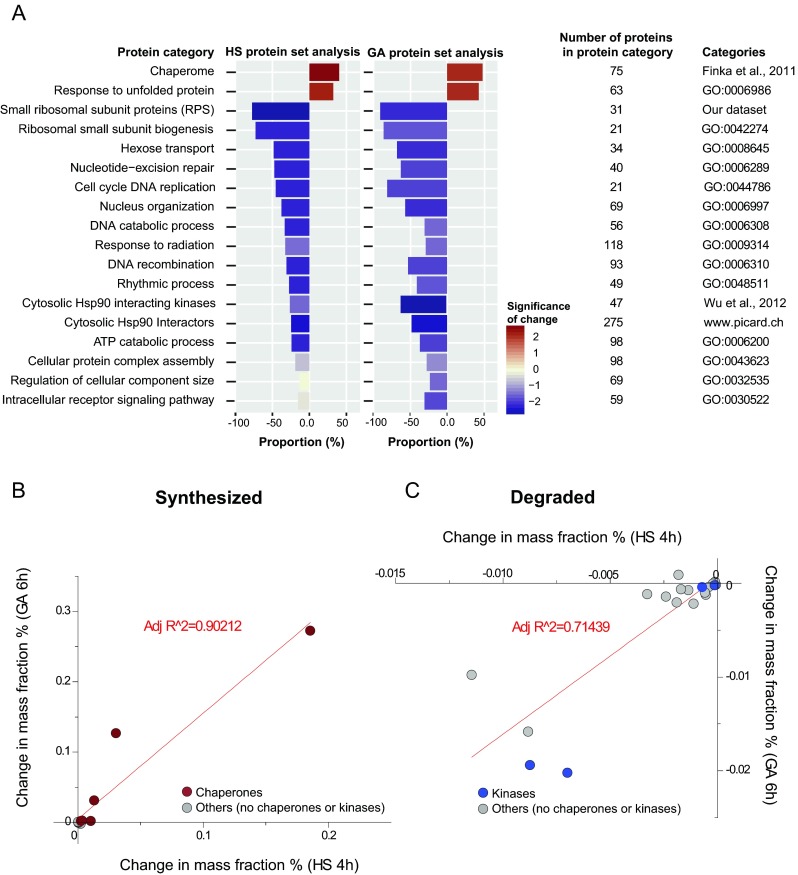
Heat and geldanamycin treatment show similar profiles of protein accumulation and depletion. **a** Categories of the most upregulated (positive numbers: *red*) and the most downregulated (negative numbers: *blue*) proteins in HS and GA treatments sorted according to their statistical significance (false discovery rate of change; *dark*: high significance, *light*: low significance). **b** Correlation analysis between the significant (*p* < 0.1) most upregulated proteins (in mass fraction) for HS versus GA treatment. Chaperones: *dark red*. Others: *gray*. **c** Correlation analysis between the significant (*p* < 0.1) most downregulated proteins (in mass fraction) for HS versus GA treatment. Chaperones: *dark red*. Kinases: *blue*. Nonchaperones, nonkinases: *gray*

We next sorted the heat-accumulated (Fig. 3a positive numbers, red) and heat-depleted proteins (Fig. 3a negative numbers, blue) in a decreasing order of significance, according to the functional Gene Ontology (GO) categories to which they belonged (from dark to light) (Wu et al. 2010). In addition to the generally annotated GO categories, four ad hoc categories were added: the cytosolic small ribosomal subunit proteins (RPS), the human chaperome (Finka and Goloubinoff 2013), HSP90-interacting kinases (Wu et al. 2012), and HSP90 interactors (http://www.picard.ch/HSP90Int/index.php). Figure 3a shows only GO categories that contained at least 20 different proteins identified and quantified in our proteomic dataset. As expected, chaperome proteins that also belonged to the canonical GO category of “unfolded protein response” were collectively upregulated. Many housekeeping proteins were among the proteins that were more depleted during HS, such as the ribosomal proteins, and also proteins involved in nucleotide-excision repair, DNA recombination, and cell cycle DNA replication as well as in Hsp90-interacting cytosolic kinases and proteins involved in nucleus organization, in agreement with the heat-induced growth arrest that we observed (Fig. 3a, left).

### Heat and geldanamycin treatments produce similar proteomic expression patterns

Microarrays (Finka et al. 2011), classic ^3^H- and ^35^S-methionine labeling (Ritossa 1962; Tissieres et al. 1974), and Western blots (Kiang et al. 1994) have shown that the heat-induced expression of HSPs, in particular of HSP chaperones, can be in part mimicked by isothermal pharmacological treatments with the HSP90 inhibitors, such as geldanamycin (GA) or radicicol (Saidi et al. 2009; Schulte et al. 1998; Sharma et al. 2012; Whitesell et al. 1992). This was confirmed in high-throughput SILAC MS proteomic studies on the effect of a 6-h treatment with a geldanamycin analogue on HeLa and Jurkat cells, which showed the upregulation of chaperome members and the apparent degradation of protein kinases and of proteins involved in the DNA damage response (Fierro-Monti et al. 2013; Sharma et al. 2012). Once the raw iBAQ values from two published drug treatments (Fierro-Monti et al. 2013; Sharma et al. 2012) were converted into relative mass fractions per cell, we compared the net mass differences in the 50 most heat-accumulated and 50 most heat-depleted proteins (as in Fig. 2) to the calculated and net mass differences in the same proteins following GA treatments in HeLa and Jurkat cells. Although many GA data were with low statistical significance owing to an insufficient number of biological repeats, similar mass fraction patterns were generally observed: Few proteins, many of which HSP chaperones, became massively accumulated by GA treatment, alongside an apparent mild across-the-board depletion of many housekeeping proteins (Supplementary Fig. 3). When sorted according to GO categories with decreasing degrees of significance (Fig. 3a, right), there was a strong overlap between the net mass differences in the HS and the GA up- and downregulated proteins. In Jurkat cells, the net mass increments of the 12 most significantly heat-accumulated proteins, half of which molecular chaperones, strongly correlated (*R* ^2^ = 0.90) with the net mass increase of the same proteins in the GA treatment (Fig. 3b, Supplementary Table 4). Similarly, the net mass change of the 19 most significantly heat-depleted proteins, which included four kinases and lacked chaperones, correlated rather well (*R* ^2^ = 0.71) with the net mass decrease of the same proteins in the GA treatment (Fig. 3c, Supplementary Table 4). Together, this confirms that in quantitative and qualitative terms of the protein accumulation and depletion, specific drugs can replace, at least in part, fever or a natural HS.

### Quantification of mRNA during heat shock

To gain knowledge on the copy number per Jurkat cell of specific mRNAs encoding for proteins that either accumulated, became depleted, or remained constant during the HS, we used the nCounter® analysis system and digitally quantified 42 specific mRNA transcripts at 15 and 30 min and 1, 2, and 4 h HS and also 2 and 6 h after the HS. Signal outputs were obtained from individual samples containing 100 ng total RNA extracted each from 10,300 cells (implying an average of 10 pg total RNA per cell), thus providing a reasonable estimate of amounts for each gene. Data were collected for mRNAs encoding for 26 chaperome members, 7 ribosomal proteins, 5 glycolytic enzymes, and 4 cytoskeletal proteins (Supplementary Table 5), which were respectively among the most abundant heat-accumulated proteins, the most heat-depleted proteins (Fig. 2a), or most heat-stable proteins (Supplementary Table 2).

**Fig. 4:**
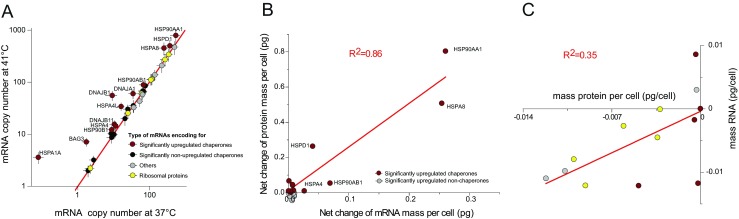
Heat-induced protein accumulation but not depletion correlates with changes in mRNA amounts. **a** Net amounts in the mRNA copies per cell of 37 selected genes encoding for significantly heat-accumulated, heat-invariable, or heat-degraded proteins following 4 h heat shock, compared to 37 °C (Supplementary Table 5). *Red median line*: no significant change, *red circles*: significantly upregulated chaperones, *black circles*: invariable chaperones. *Yellow circles*: ribosomal proteins, *gray circles*: others. **b** Correlation between the net changes in mass of 11 significantly heat-upregulated mRNAs (Supplementary Table 4) and the net changes in their corresponding protein mass. *Red circles*: significantly upregulated chaperones. *Gray circles*: significantly upregulated nonchaperones. **c** Correlation between the net change in mass for 13 significantly downregulated mRNA (Supplementary Table 4) and the net change in their corresponding protein mass (*red circles*: chaperones, *yellow*: ribosomal proteins, *gray circles*: others)

When comparing the total mRNA copy numbers of all the significantly detected members of this list at 37 °C, as compared to 41 °C, 12 genes were found to significantly depart above the average. Not unexpectedly, they turned out to be 12 chaperome members, which presented significantly higher mRNA copy numbers during HS (Fig. 4a, red dots). Noticeably, none of the mRNA copy numbers of the significantly unchanged or depleted proteins were found below the median, indicating that, in general, all mRNAs remained stable during the HS (Fig. 4a, gray, black, and yellow dots). A strong correlation (*R* ^2^ = 0.86) was observed between the net mass increase of each of the 12 most abundant heat-accumulated proteins and the net mass increase of their corresponding heat-accumulated mRNAs (Fig. 4b). In contrast, a weak correlation (*R* ^2^ = 0.35) was found between the net mass decrease of the heat-depleted proteins and the net mass changes of their corresponding mRNAs (Fig. 4c). Thus, whereas HSP accumulation could be attributed to an increase of their corresponding mRNAs, the mild depletion of many housekeeping proteins could not be attributed to the degradation of their mRNA.

**Fig. 5:**
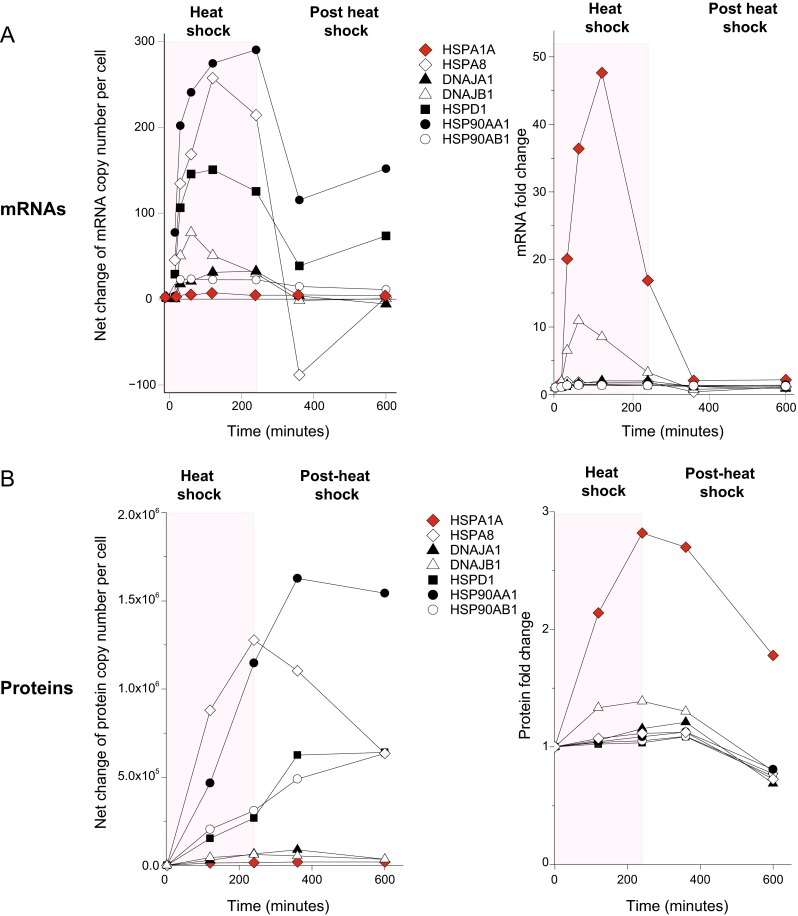
Time-dependent absolute and relative changes in specific mRNA and protein amounts during and after heat treatments. **a** Changes in net number of mRNA copies per cell, encoding for the heat-upregulated proteins HSPA1A, HSP8, DNAJA1, DNAJB1, HSPD1, HSP90AA1, and HSP90AB1. *Left*, during 4 h heat shock at 41 °C (*pink background*) and up to 6 h at 37 °C after the heat shock (*white background*), and their corresponding values expressed in “fold changes” (*right*). **b** Changes in net protein number per cell as in **a** (*left*) and their corresponding values expressed in “fold changes” (*right*)

The net changes in the cellular amounts of the mRNAs of HSPA8, HSPA1A, HSP90AA1, HSP90AB1, HSPD1, DNAJA1, and DNAJB1 were monitored in time during and following HS and expressed in net copy number differences (Fig. 5a, right), instead of classically in “fold changes” relative to the basal level at *T* = 0 (Fig. 5a, left). At unchallenging 37 °C, the HSPA1A mRNA level was barely above the detection threshold with less than a single mRNA molecule in ten cells and it increased to ~5 mRNA per cell at 2 h HS. This translated into an impressive, yet misleading, 49-fold apparent increase in mRNA amount (Fig. 5a). As with HSPA1A, a more than 2-fold upregulation was observed for BAG3, DNAJA1, and DNAJB1, which all had very low absolute mRNA copy numbers at 37 °C (Supplementary Fig. 3A). In the case of HSPA6 mRNA, the level was below detection at 37 °C. It increased to a mere single copy per cell following 4 h at 41 °C, pointing at the importance of expressing this data in net differences of mRNA copies, rather than in “fold changes, to properly reflect an ongoing biological process.

Confirming that HSPs are in general much more stable than their mRNAs, the mRNA of most HSPs rapidly degraded following the HS, whereas their corresponding proteins remained stable or even continued to accumulate for a while after the HS. This was observed in the case of HSP90AA1, whose net protein/mRNA ratio was ~3900 at 4 h HS, but owing to the rapid mRNA degradation and a concomitant protein accumulation, evolved into a ratio of 14,500 at 2 h after the HS (Fig. 5a, b, right panels). This furthermore illustrated the great difficulty of choosing a single time point at which mRNA and protein levels may provide a fair representation of a given cellular response, such as HS. Instead, a suitably calibrated combination of proteomic measurements at multiple time points, as we performed here, is needed to faithfully serve as a hallmark of cellular physiology.

### Absolute changes in copy numbers best estimate the kinetics of mRNA and protein synthesis and depletion

In contrast to HSPA1A/B (HSP70), the steady-state mRNA levels of HSPA8 at 37 °C were high, and although generally considered unresponsive to heat stress (Ingolia and Craig 1982), HSPA8 mRNA was found to nearly double during the 4-h HS, corresponding to a net gain of 240 mRNA molecules per cell (Fig. 5a, left). This translated into a net gain of 1,300,000 new HSPA8 polypeptides per cell (Fig. 5b, left), which, when expressed in fold changes, was a mere 1.17-fold increase (Fig. 5b, right). In contrast, the mRNA copies of the heat-induced isoform of the chaperone, HSPA1A, apparently increased 49 times in 2 h HS (Fig. 5a, right), but in real numbers formed about 5 new mRNA copies per cell that produced 24,000 new HSP70 polypeptides per cell (Fig. 5b, right). Thus, high-throughput proteomics revealed a paradoxical situation whereby during HS the heat-inducible isoform HSP70 accumulated 55 times fewer polypeptides than its presumably nonheat-inducible isoform, HSPA8 (HSC70).

## Discussion

### The biological relevance of quantitative HSP expressomics

The chaperome network, which can accumulate even without stress into 10 % of the total protein content of human cells (Finka and Goloubinoff 2013), controls protein homeostasis in cellular growth, differentiation, steady-state functions, defenses, stress management, and programmed cell death (Brehme et al. 2014; Bromberg et al. 2013; Finka et al. 2012; Kim et al. 2013; Weiss et al. 2007). In stressed aging mammalian neurons, the onset of degenerative protein misfolding diseases, such as spinocerebellar ataxia, Parkinson and Alzheimer’s diseases, tightly correlates with a general decrease of chaperone concentrations and implies alterations of the stoichiometries in the chaperome network and the impairment of proteostasis in general (Yang et al. 2014). Moreover, in old diseased cells, many chaperones that do not degrade are found abnormally associated to insoluble protein aggregates where they are apparently inactive. On the contrary, in cancer cells, apoptosis is apparently blocked by abnormally elevated chaperone concentrations expressed even at non-HS temperatures, in correlation with increased cell survival to aggressive chemotherapies (Bromberg et al. 2013; Finka and Goloubinoff 2013; Hinault et al. 2006; Wang et al. 2014).

Acquired thermotolerance is a short-term ability of all organisms, bacteria and eukaryotes alike, to survive otherwise short deadly exposures to extreme HS (Finka et al. 2012; Mittler et al. 2012). In all organisms, a mild prior, so-called priming HS, which can timely induce the accumulation of HSPs, HSP chaperones in particular, is a prerequisite to establish effective acquired thermotolerance (Larkindale and Vierling 2008). A qualitative and precise quantitative assessment of the specific HSPs that need to accumulate in a timely fashion above a given baseline level, or of proteins that need to be depleted during the HS priming, is central to the understanding of the molecular mechanisms conferring thermotolerance to stressed organisms and, importantly, also conferring resistance of cancerous cells to thermo-, chemo-, or radiotherapies (Rylander et al. 2005). Here, using SILAC and quantitative mass spectrometry, we addressed the identity and estimated the cellular quantities of proteins that become either significantly accumulated or depleted in human cultured cells, during and following a short mild heat shock at 41 °C.

### Mild heat shock causes a global protein mass change of about 2 %

Our methodology to convert raw iBAQ values into relative mass fractions and absolute copy numbers per constant cell volume (Finka and Goloubinoff 2013) provided individual protein concentrations that were in good agreement with independent estimates of cellular densities of ribosomes (Duncan and Hershey 1983) and histones (Winiewski et al. 2014) in human cell cultures. The major limitation of our medium-depth high-throughput proteomic analysis was the need to perform many costly independent biological replicates to statistically cover in our analysis at least 90 %, mass-wise, of the cellular proteins.

Our analysis showed a total approximate net mass increment of 2 % in 4 h HS, more than two thirds of which (1.4 %) were significantly contributed by the chaperome members HSP90AA1, HSPA8, HSP90AB1, HSPD1, and HSPA5. This mass increase was counterbalanced by an apparent across-the-board mild depletion of several hundred proteins, many of which with housekeeping functions, such as the ribosomal proteins, the cytosolic HSP90-interacting kinases, proteins involved in the cell cycle (Kuhl and Rensing 2000), and proteins involved in nucleus organization and DNA metabolism (Sharma et al. 2012).

Protein depletion unlikely resulted from the degradation of their corresponding mRNAs, as none of the tested mRNA species were significantly degraded by heat. As well, protein degradation unlikely resulted from the heat-induced activation of proteases, because the mild across-the-board protein depletion that was observed during the HS was maintained for a few hours after the HS, and the same proteins that were depleted during the HS were also depleted to similar extents by an isothermal geldanamycin treatment. Instead, several effects could contribute to the mild across-the-board depletion of many proteins during HS, without any decrease of their corresponding mRNA. Earlier reports (Fierro-Monti et al. 2013; Laramie et al. 2008) have shown that during HS, the mRNA of housekeeping proteins may become stabilized within heat-shock granules (Kedersha et al. 1999). It may thus remain sequestered away from ribosomes as long as the HS continues (Lavut and Raveh 2012). Furthermore, the limited pool of cytosolic ribosomes, which we found to be mildly (~10 %) depleted during the HS, was also recruited by the newly synthesized HSP mRNAs. Ribosomal recruitment by an excess of HSP mRNAs is expected to transiently slow down the steady rate at which most housekeeping proteins are being replenished. In addition, HS has also been shown to slow down translation in general (Shalgi et al. 2013). Thus, owing to the specific sequestration of the mRNAs encoding for house-keeping proteins in stress granules, their being outcompeted in part by the newly accumulated HSP mRNAs for slightly fewer ribosomes, and the general slowing down of translation, the mild across-the-board depletion of many housekeeping proteins during HS may be attributed to their slower replenishment, rather than to increased protein degradation per se.

Given that heat induces fluidity changes and the transient opening of specific Ca^2+^ entry channels in the plasma membrane (Bromberg et al. 2013; Finka et al. 2012; Saidi et al. 2009), how may geldanamycin induce a response similar to HS? Although this is unclear, the geldanamycin HS-like isothermal effects on mammalian cells, as well as the radicicol isothermal effects on plant cells, have been clearly demonstrated to similarly depend on the entry of extracellular Ca^2+^ through specific Ca^2+^ conducting channels in the plasma membrane (Saidi et al. 2009; Sun et al. 2013). Early studies involving the coinjection of denatured proteins with Hsp genes into frog oocytes showed the isothermal activation of the Hsp genes, whereas coinjected native proteins did not (Ananthan et al. 1986). It is tempting to speculate that the acknowledged aggregation of geldanamycin-induced released clients from Hsp90 would generate artefactual hydrophobic interactions with membranes resulting in a similar membrane destabilization effect and a HS-like response.

### What are the main HSPs and how may they contribute to thermotolerance and survival?

It should be noted that our results were obtained after a mild stress (41 °C), corresponding to typical heat-priming conditions for effective acquired thermotolerance. However, stronger stresses, such as 43 or 45 °C, may result in dramatically different profiles of protein accumulation and degradation (De Maio et al. 1993). Not unexpectedly, our quantitative proteomic analysis from 41 °C-treated cells revealed that out of the seven most heat-accumulated proteins, five were core members of the cytosolic chaperome network (Finka et al. 2011) that can all use ATP to perform work on protein substrates: the two main HSP90s (HSP90AA1 and HSP90AB1), the most abundant HSC70 (HSPA8), and two HSP70 cognates (HSPA4 and HSPA1A). HSP60, the mitochondrial orthologue of bacterial GroEL (HSPD1), was the main heat-induced mitochondrial protein and 70 kDa BiP (HSPA5) and 90 kDa GRP94 (HSP90B1, endoplasmic) were the major HS-accumulated ER proteins. As initially shown from relative mRNA expression data, the quantitative proteomic data confirmed that the heat-shock response is primarily taking place in the cytoplasm and the mitochondria, whereas the ER proteins remain relatively unchanged, although under a reducing stress, ER chaperone genes can specifically accumulate and cause a typical expression pattern commonly referred to as the unfolded protein response (Maly and Papa 2014). It should be noted that even for the most heat-accumulated chaperones, namely HSP90AA1, HSP90AB1, and HSC70, the net mass increments were, respectively, 8, 5, and 11 %, compared to their basal elevated cellular concentrations under unstressed conditions. Our choice to show here the absolute net differences of HSP mass per cell, rather than expressing them as a “fold change” ratio, raises the question of how, e.g., an increase of “only” 8 % in cytosolic HSP90 might make the difference between dying or surviving to a noxious heat stress? Noticeably, the J-domain co-chaperones that specifically target misfolded substrate proteins onto the HSP70s and HSP110s (Hinault et al. 2010; Mattoo et al. 2013) and the various regulators/co-chaperones of HSP90 (http://www.picard.ch/HSP90Int/index.php) are present in the cell in much lower amounts. Consequently, with the exception of DNAJA1, DNAJB1, and STIP1, chaperone concentrations and their possible variation under HS may have remained below the significance threshold of our analysis. The minor copy number differences in various co-chaperones, such as DNAJA1, DNAJB1, and BAG3, as also suggested from microarray analysis (Finka et al. 2011), could strongly modify the specificity and efficacy of the chaperone network at repairing key heat-damaged proteins. Taken together, the net gain of about 1.4 × 10^6^ HSP90s and 1.3 × 10^6^ HSC70 in the cytosol could establish a transient thermotolerance phenotype and prevent heat-induced cell death by mechanisms that still need experimental investigation. A more refined in-depth proteomic analysis, with more biological replicates in order to increase depth, is also needed to resolve minor variations among the main regulatory co-chaperones, to understand how mild relative quantitative changes in the ATP-hydrolyzing core chaperone machineries could change the fate of sensitive human cells to become thermotolerant, or resist aggressive chemotherapies.

### Possible functional differences between HSC70 and HSP70

Classic mRNA microarray analysis and Western blots have established that in unstressed human cells, cytosolic HSPA1A/B (HSP70) is generally expressed at very low basal levels, whereas it strongly accumulates during and following HS (Beere 2005). In contrast, cytosolic HSC70 (HSPA8), which shares 90 % amino acid identity with HSPA1A/B, is expressed under unstressed conditions at a constant elevated level that remains virtually unchanged during and following stress (Finka et al. 2011). Noticeably, most types of aggressive cancer cells resisting chemotherapies express abnormally high constitutive levels of HSPA1A/B without HS (Ciocca and Calderwood 2005; Laramie et al. 2008). High HSPA1A/B levels in cancer cells serve as hallmarks for poor survival prognostics (Kaur et al. 2011). Thus, HeLa and HEK293 cells may accumulate astounding concentrations of HSPA1A/B without stress, respectively, 0.5 and 1 % of their total protein mass (Supplementary Fig. 4) (Finka and Goloubinoff 2013). Our choice to focus on Jurkat cells was guided by the relatively low constitutive expression levels of HSPA1A/B as compared to HSPA8. Despite being transformed human cells, Jurkat cells were thus the best paradigm next to unchallenged human naive cells. There is a large body of evidence in plant and animal tissues that the specific overexpression of HSPA1A/B, and not of HSC70, has a strong antiapoptotic effect. Thus, the specific overexpression of HSPA1A/B prevents apoptosis in Jurkat cells by blocking the caspase pathway through APAF-1 and procaspase-9 (Beere et al. 2000; Pandey et al. 2000) and also by a caspase-independent mechanism involving the association of HSPA1A/B to the apoptosis protease activating factor-1 (Ravagnan et al. 2001). The transient overexpression of an active form of HSPA1A/B by way of an adenovirus in the lungs of ARDS rats can specifically arrest sepsis-induced apoptosis in alveolar type-1 cells, thereby greatly increasing animal survival (Weiss et al. 2007). Interestingly, using domain swaps between primate as well as plant Hsp70s and HSC70s, it has been elegantly shown in yeast that HSC70 is the predominant species involved in growth and survival to HS (Tutar et al. 2006). Similarly, in higher plants, the transient overexpression of HSPs during a mild priming heat shock can effectively prevent apoptosis caused by a subsequent noxious heat shock (Finka et al. 2012), suggesting that in all eukaryotes in general, the timely accumulation of HSPs, especially of HSPA1A/B, may be a means to transiently inhibit deleterious stress-induced apoptotic signals (Zorzi and Bonvini 2011). However, since in the long term apoptosis is central to tissue differentiation and for animals to combat cancer, this blockage would need to be short-lived (for a review, see Elmore 2007). In this context, we and others have observed that the new heat-induced HSPA1A/B and DNAJB1 mRNAs were unstable and started to degrade already within 1 h of the HS, already during the ongoing HS (Fig. 5) (Doong et al. 2003; Gotoh et al. 2001, 2004), as first observed by Theodorakis and Morimoto (1987). Such exceptional degradation of the HSP70 mRNA could ensure in the medium term the restoration during and after the stress the essential apoptotic pathway, which would not occur in resistant cancer cells.

The unexpected two orders of magnitude difference that we found between the massive net copy number of heat-accumulated HSC70 and the minor heat-accumulated HSPA1A/B species suggests that HSC70 likely acts more as a generalist chaperone preferably targeting proteins misfolded by the HS and uses a mechanism of ATP hydrolysis-driven *ultra-affinity* (De Los Rios and Barducci 2014) to tightly bind and prevent their aggregation with high efficiency (Sharma et al. 2011). Only once the heat stress is over, the partially unfolded HSP70-bound polypeptide substrates (Sharma et al. 2010) may be released and allowed to refold to the native state, or when irreversibly damaged, it may be targeted to proteasomal or lysosomal degradation (Hinault et al. 2006). Moreover, HSC70, which we found to be as abundant as the ribosomes, could participate as members of the RAC/NAC complex (Koplin et al. 2010) and thereby apply at the exit of the heat-shocked ribosomes an entropic pulling force (De los Rios et al. 2006) to resume the progression of stalled nascent chains in the ribosomal channel (Liu et al. 2013; Shalgi et al. 2013).

In contrast, the newly synthesized HSPA1A/B molecules, which we found to be about 55 times fewer than the newly synthesized HSC70s, unlikely act as a generalist chaperone. Its minute amounts would rather fit a specialized function such as targeting specific native proteins, such as Apaf1 and IκB (Aschkenasy et al. 2011; Sabirzhanov et al. 2012; Weiss et al. 2007) of the apoptosis signaling pathway. Upon binding, HSPA1A/B could locally unfold and thus inactivate their native substrates during the stress and release them after the stress into natively refoldable species capable to resume the signal transduction, including apoptosis. Another possibility would be that the inducible isoform, HSPA1A, is specific for locating into special cell compartments and thus attaining a much higher local concentration, but at the same time maintaining the ability to act as a generalist in these special compartments. An example would be the localization of HSPA1A to “dynamic droplets” (reviewed in Amen and Kaganovich 2015).

### Quantitative proteomic analysis for diagnostic purposes

Transcriptomic mRNA profiles have been used to identify genes specifically expressed at particular stages of tissue development or under various challenges, including drugs, physical stimuli, pathogens, and pathologies such as aging and cancer (Aiba et al. 2006; Chibon 2013; Eddy et al. 2010; Weeraratna 2005). These mRNA profiles, which can be found in various databases and in web-based applications such as Genevestigator (Hruz et al. 2008), are nevertheless generally expressed in relative “fold change” ratios, which can generate misleading interpretations because of the default division by insignificantly low values that are often variable. In addition, the presence of mRNA is not a corollary for a biologically relevant activity, as for example, mRNAs can be sequestered in heat-shock granules or stalled at a given position on a ribosome, as shown by ribosomal profiling during HS to be the case of many housekeeping mRNA (Shalgi et al. 2013). In contrast, heat-shock proteins, which ultimately carry out the heat stress-defensive/protective functions, are generally more long-lived than their mRNAs. This is exemplified by HSP70 and HSP90 in plant and animals, whose transcripts rapidly accumulate during the first hour of a continuous mild HS, to thereafter rapidly decay and reach near basal levels within 24 h despite the ongoing HS (Finka et al. 2012). In contrast, the consequent new amounts of HSP70s and HSP90s start decaying at a slower pace than the mRNAs, much later in the HS (Fig. 5). Thus, for diagnostic purposes, quantitative MS proteomics of net absolute mass changes (not of relative fold mass changes) likely provide more meaningful outputs. Even unlabeled proteomic analysis of unstressed cancer cells for example can provide rather precise quantities for a few hundreds proteins, such as HSPs and metabolic enzymes, that could serve for diagnostic purposes, as in the case of particularly resistant cancer cells displaying abnormally high levels of HSP70 and high levels of metabolic enzymes involved in glycolysis (Finka and Goloubinoff 2013). The great similarities of net mass differences of specific proteins, which we found between GA- and HS-treated cells, suggest that quantitative proteomics could also serve to identify new drugs capable to specifically elicit changes in the cellular concentrations of particular protein sets, such as the molecular chaperones, in search for therapies against degenerative protein conformational diseases.

## Electronic supplementary material

Below is the link to the electronic supplementary material.

Supplementary Fig. 1 Mean of change in mass fraction of 50 most incremented and decremented proteins, computed as in Fig. 2a, at *T* = 120, 240 during HS and at 360 and 600 min following the HS. At all time points of the experiments, few HSPs massively accumulate. (PDF 242 kb)

Supplementary Fig. 2 (**A**) Bar graphs showing net increase and decrease of cumulative mass fraction changes in heat-depleted (blue left wise) and heat-accumulated proteins computed in decreasing bins of significance as in Fig. 2b, at *T* = 120, 240 during HS and at 360 and 600 min following the HS. (**B**). At all time points of the experiments, more proteins are mildly depleted while a few HSPs massively accumulate. As in Fig. 2c, values were separately calculated for *T* = 120, 240, 360 and 600 min. (PDF 185 kb)

Supplementary Fig. 3 Bar graphs showing net increments and decrements of protein mass fraction that are caused by geldanamycin in Jurkat and HeLa cells. Mean of change in mass fraction of 50 proteins being the most incremented (upper graph) and decremented (lower graph) by HS as in Fig. 2a. Mean net significant change in the mass fraction (%) of the 50 most heat accumulated (red) and depleted proteins (as in Fig. 2a, compared to 6 h in the presence of geldanamycin at 37 °C and of the 50 most heat depleted proteins (blue) at all time points). (PDF 353 kb)

Supplementary Fig. 4 HSPA8 and HSPA1A amounts in unstressed cancer and naïve human cells. Absolute quantities in percent of total protein mass of HSPA8 (Hsc70, black bars) and HSPA1A (Hsp70, red bars) in eleven immortalized human cell cultures (Geiger et al. 2012) showing nearly constant levels of HSPA8, as compared to highly variable levels of constitutively expressed HSPA1A. Unlike most cancerous cell lines, the Jurkat cells are observed to reproduce most typically the expression profile of unchallenged total naïve human cells in which HSPA1A levels are maintained low. (PDF 282 kb)

Supplementary table 1 Raw iBAQ data containing 4’585 detected polypeptides in Jurkat human cells that were either continuously at 37 °C as controls or heat-treated at 41 °C for 2 and 4 h and thereafter for 2 and 6 h again at 37 °C. This dataset is thus composed of 24 independent biological samples of treated and control triplicates at four time points (Fig. 1) : a triplicate at 2 h 41 °C, a control triplicate at 2 h 37 °C, a triplicate at 4 h 41 °C, a control triplicate at 4 h 37 °C, a triplicate at 6 h, 2 h after HS, a control triplicate at 6 h always at 37 °C, a triplicate at 10 h, 6 h after HS, a control triplicate at 10 h always at 37 °C. (XLSX 31957 kb)

Supplementary table 2 Filtered protein table containing significant 3’164 protein mass fraction at 37 °C and 41 °C. (XLSX 1214 kb)

Supplementary table 3 (**A**) Protein table containing individual protein mass fraction at 37 and 41 °C. (**B**) Protein table containing individual protein copy numbers at 37 and 41 °C (XLSX 3984 kb)

Supplementary table 4 List of significantly accumulated and depleted proteins following a 4 h HS treatment at 41 °C (this study), as compared published proteomic data from Jurkat cells treated isothermally for 6 h with the HSP90 inhibitor, geldanamyicin (44). (XLSX 11 kb)

Supplementary table 5 The copy number per Jurkat cell and raw NCounter data of 42 mRNAs encoding for a chosen subset of glycolytic enzymes, molecular chaperones, cytoskeletal and ribosomal proteins, at 37 and 41 °C at the different time points of the experiment, as indicated. (XLSX 50 kb)
