# Supplementary figures and images for: Quantitative proteomics of heat-treated human cells show an across-the-board mild depletion of housekeeping proteins to massively accumulate few HSPs
Source: Cell Stress Chaperones. 2015 Apr 8;20(4):605–20. doi: 10.1007/s12192-015-0583-2 (PMC4463922; doi:10.1007/s12192-015-0583-2)

# Supplementary figure 1

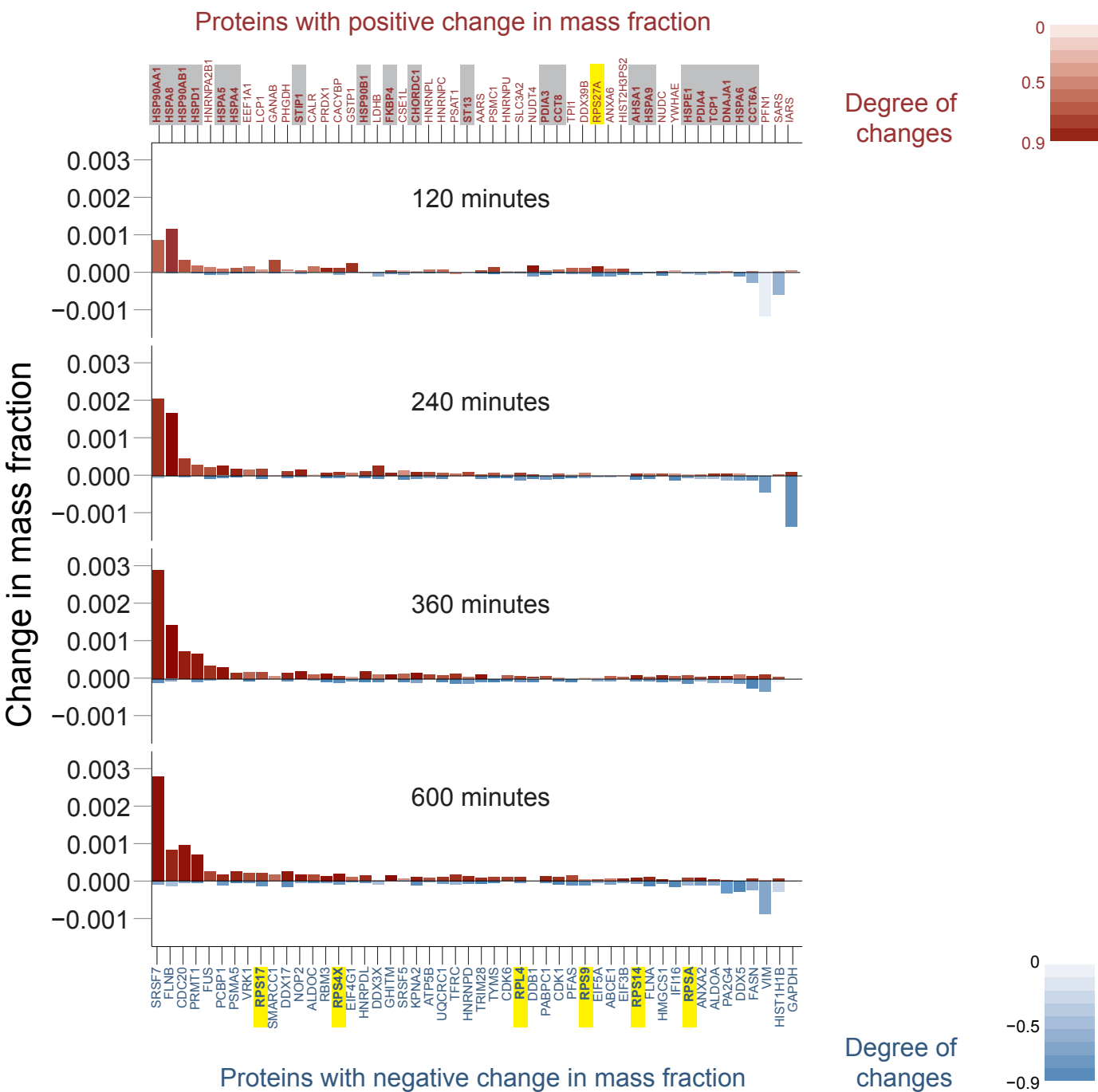

Supplement: Supplementary file 1 — Mean of change in mass fraction of 50 most incremented and decremented proteins, computed as in Fig. 2a, at T = 120, 240 during HS and at 360 and 600 min following the HS. At all time points of the experiments, few HSPs massively accumulate. (PDF 242 kb) [file 12192_2015_583_MOESM1_ESM.pdf]

Supplementary figure 2

A

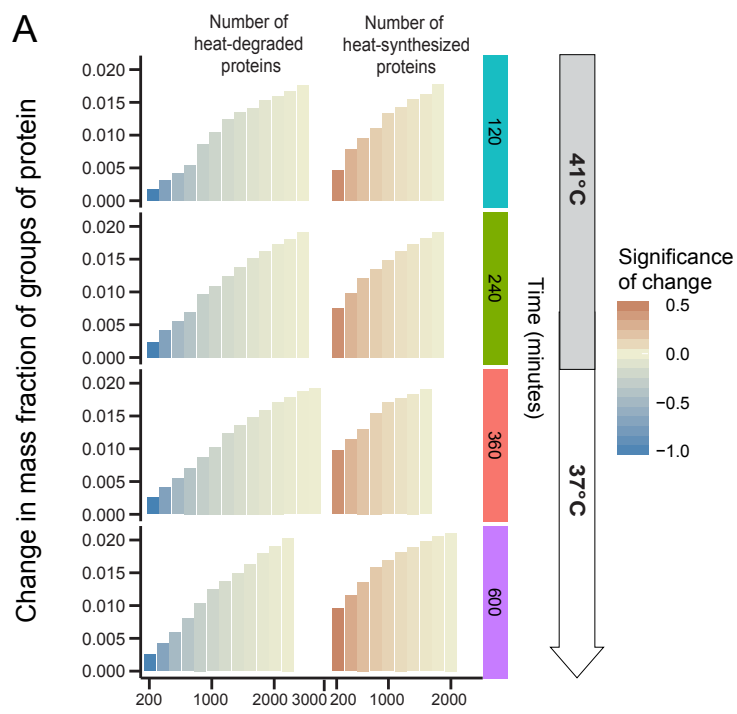

B

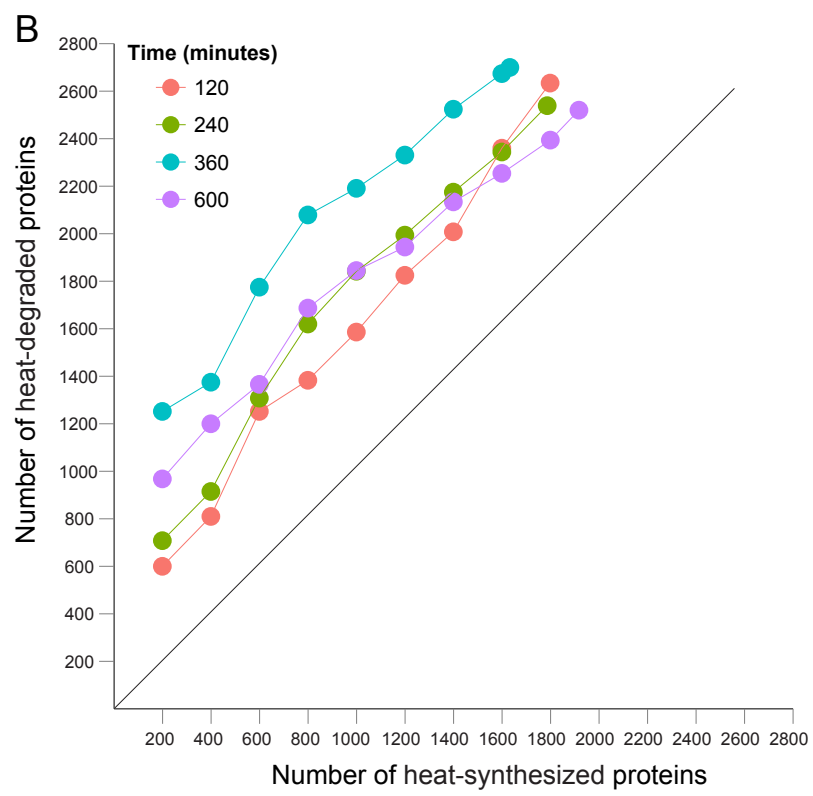

Supplement: Supplementary file 2 — (A) Bar graphs showing net increase and decrease of cumulative mass fraction changes in heat-depleted (blue left wise) and heat-accumulated proteins computed in decreasing bins of significance as in Fig. 2b, at T = 120, 240 during HS and at 360 and 600 min following the HS. (B). At all time points of the experiments, more proteins are mildly depleted while a few HSPs massively accumulate. As in Fig. 2c, values were separately calculated for T = 120, 240, 360 and 600 min. (PDF 185 kb) [file 12192_2015_583_MOESM2_ESM.pdf]

Supplementary figure 3

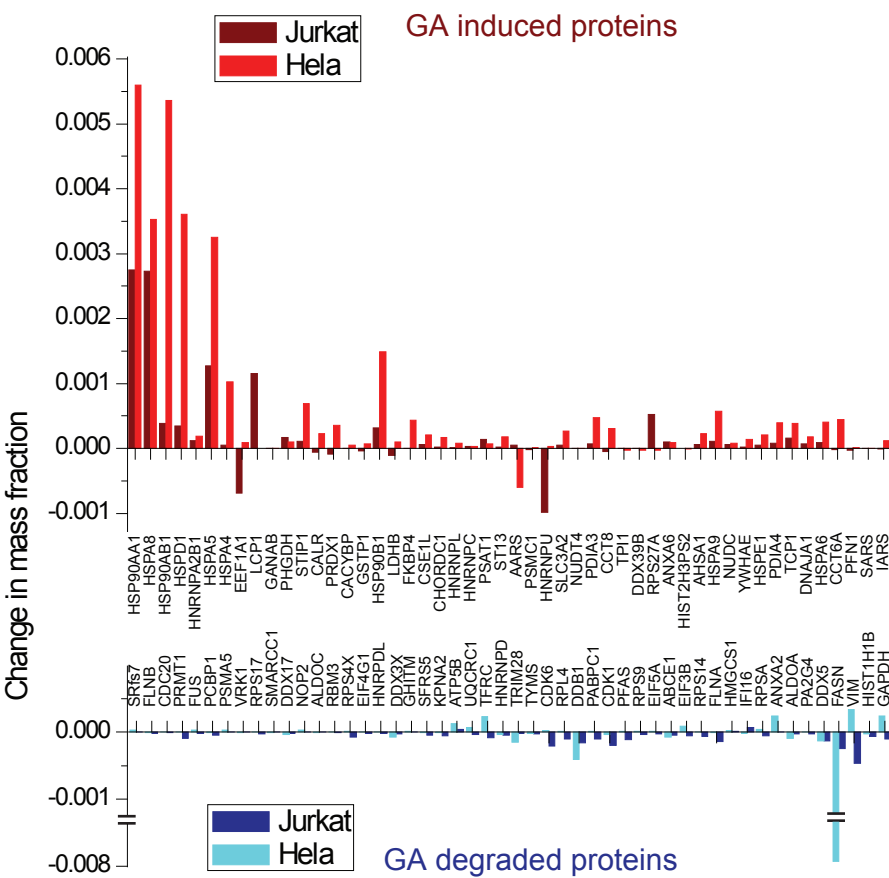

Supplement: Supplementary file 3 — Bar graphs showing net increments and decrements of protein mass fraction that are caused by geldanamycin in Jurkat and HeLa cells. Mean of change in mass fraction of 50 proteins being the most incremented (upper graph) and decremented (lower graph) by HS as in Fig. 2a. Mean net significant change in the mass fraction (%) of the 50 most heat accumulated (red) and depleted proteins (as in Fig. 2a, compared to 6 h in the presence of geldanamycin at 37 °C and of the 50 most heat depleted proteins (blue) at all time points). (PDF 353 kb) [file 12192_2015_583_MOESM3_ESM.pdf]

## Supplementary figure 4

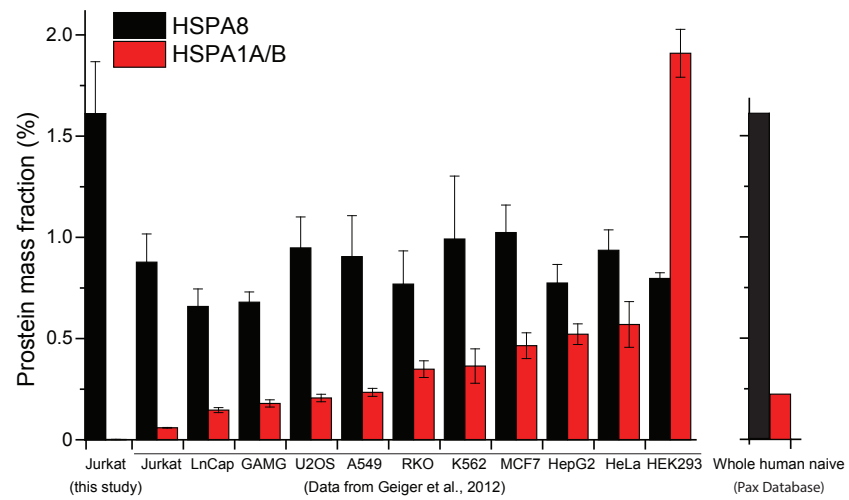

Supplement: Supplementary file 4 — HSPA8 and HSPA1A amounts in unstressed cancer and naïve human cells. Absolute quantities in percent of total protein mass of HSPA8 (Hsc70, black bars) and HSPA1A (Hsp70, red bars) in eleven immortalized human cell cultures (Geiger et al. 2012) showing nearly constant levels of HSPA8, as compared to highly variable levels of constitutively expressed HSPA1A. Unlike most cancerous cell lines, the Jurkat cells are observed to reproduce most typically the expression profile of unchallenged total naïve human cells in which HSPA1A levels are maintained low. (PDF 282 kb) [file 12192_2015_583_MOESM4_ESM.pdf]
